# Supplementary material for: Cardiopatch platform enables maturation and scale-up of human pluripotent stem cell-derived engineered heart tissues
Source: Nat Commun. 2017 Nov 28;8:1825. doi: 10.1038/s41467-017-01946-x (PMC5705709; doi:10.1038/s41467-017-01946-x)
Supplement: Supplementary file 1 — Supplementary Information [file 41467_2017_1946_MOESM1_ESM.pdf]

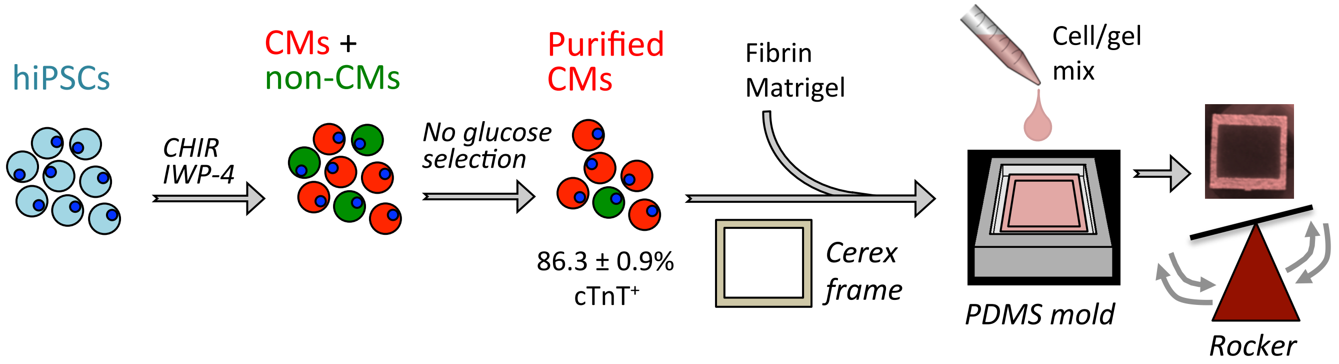

Supplementary Figure 1. Schematic of cardiopatch fabrication and culture. hiPSCs are differentiated into a mixture of cardiomyocytes (CMs) and non-CMs, then metabolically purified using no glucose selection. Resultant dissociated cells are mixed with a fibrin/Matrigel hydrogel solution and polymerized around a Cerex frame inside a PDMS mold. Once removed from molds, cardiopatches are cultured on a dynamic platform (rocker) for 3wks.

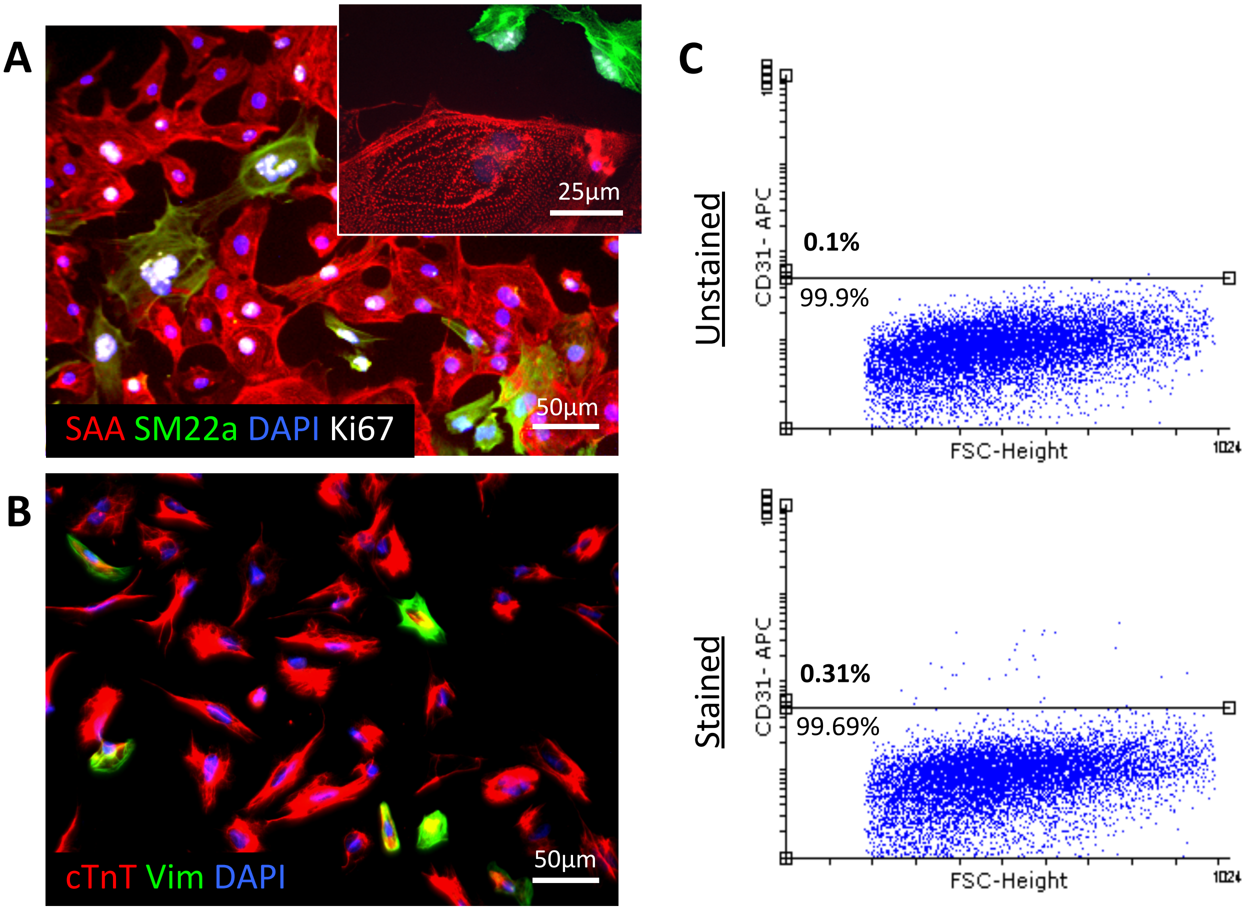

Supplementary Figure 2. **Composition of cells used for generation of cardiopatches.** A,B) Representative image of differentiated hiPSCs on d18 cultured on fibronectin for 5 days and immunostained for sarcomeric α-actinin (SAA), SM22α and Ki67 (A) and cardiac Troponin-T (cTnT) and Vimentin (Vim) (B); Inset in A showing striated hiPSC-CMs and smooth muscle cells. C) Representative flow cytometry readout of $CD31^+$ endothelial cells in population used to generate cardiopatches; Unstained (top) and CD31-APC stained (bottom) cells. Scale bar A-B) 50µm, inset 25µm.

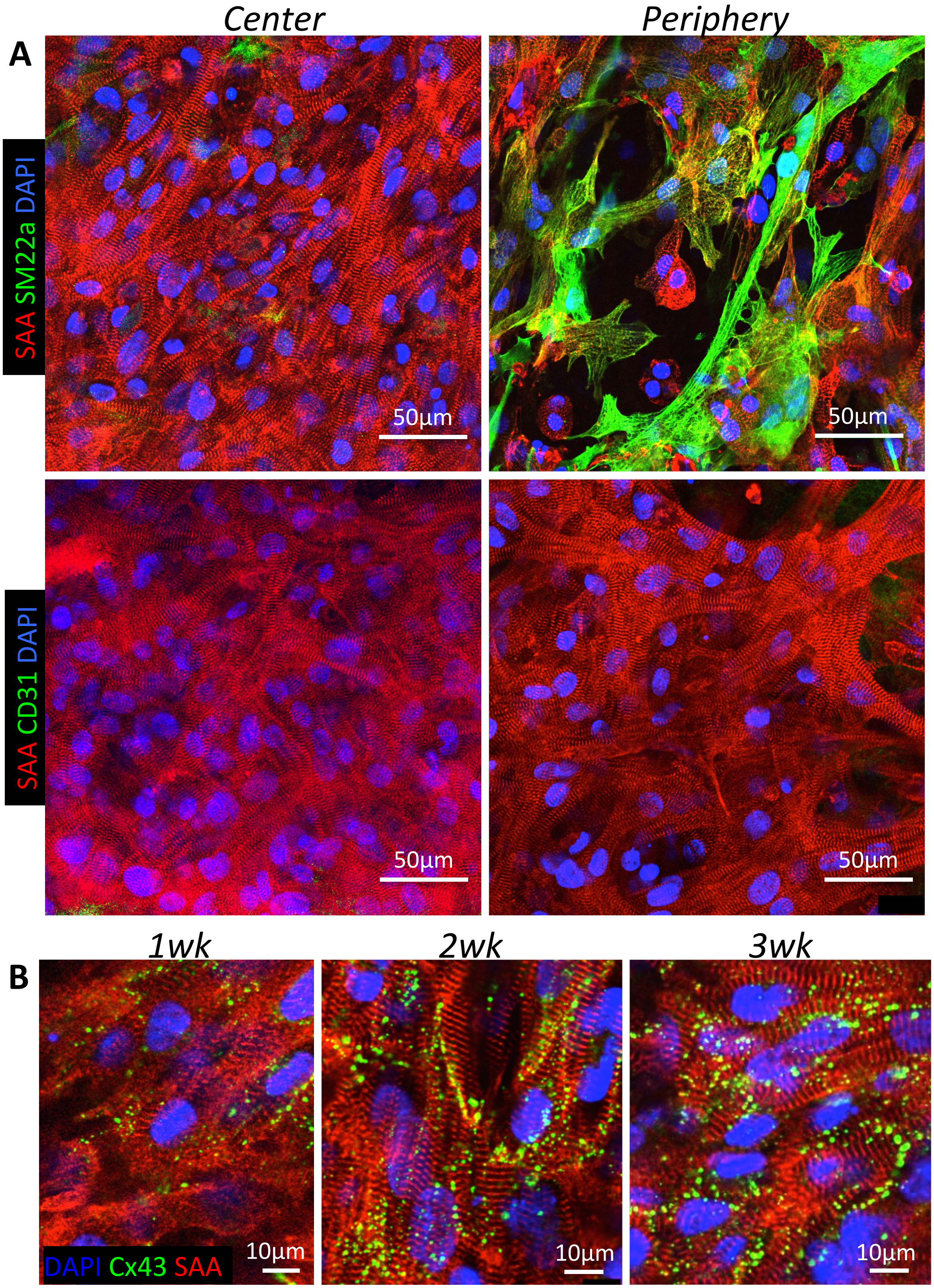

Supplementary Figure 3. Cellular composition of cardiopatches. A) Representative distributions of SAA⁺ hiPSC-CMs, SM22α^+ hiPSC-smooth muscle cells, and CD31⁺ hiPSC-endothelial cells in the center and periphery of 3wk-old cardiopatches. Note lack of endothelial cells in the patch. B) Confocal images of Cx43⁺ gap junctions in 1, 2, and 3wk-old cardiopatches. Scale bar A) 50µm, B) 10µm.

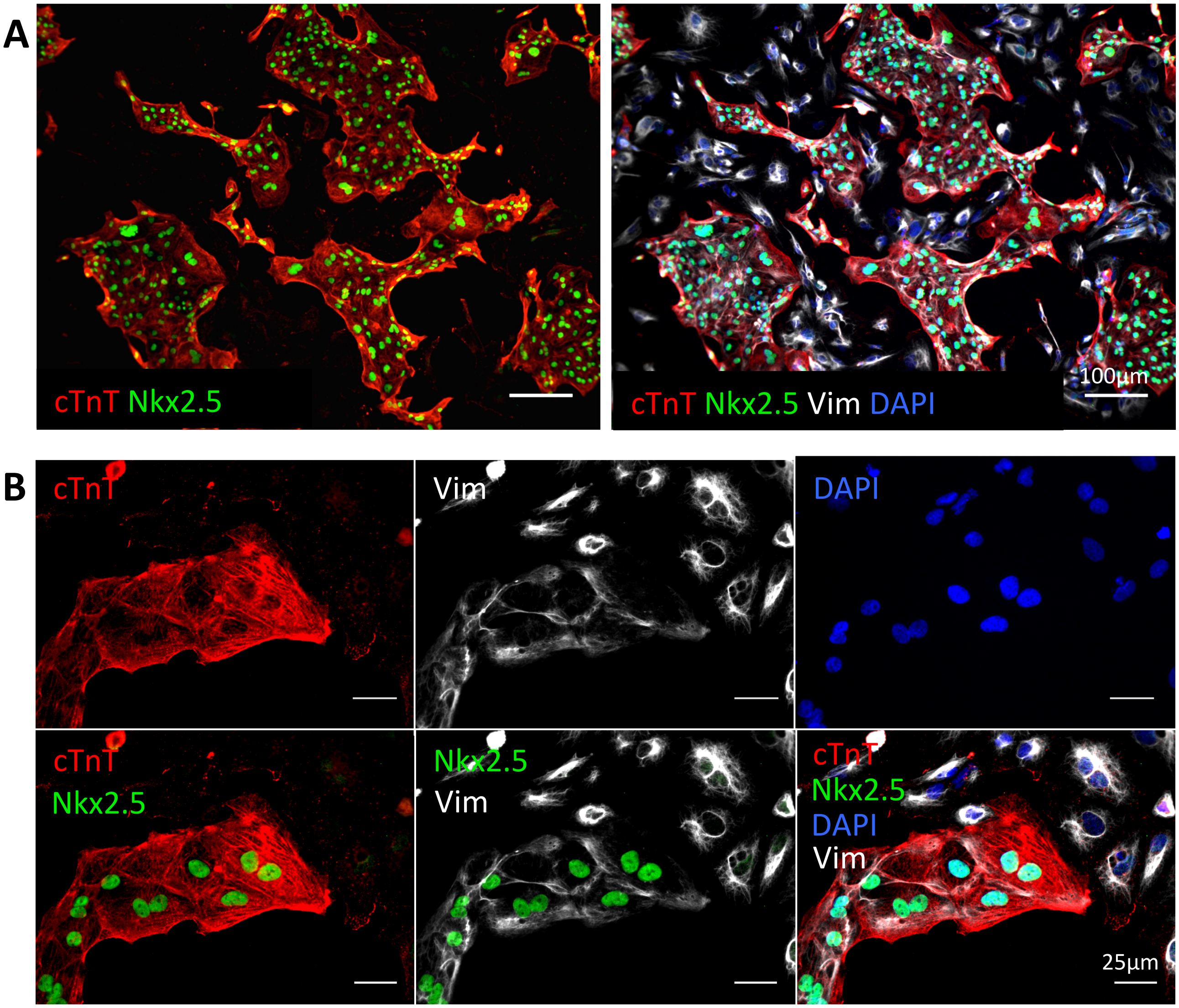

Supplementary Figure 4. Cardiomyocyte specificity of Nkx2.5 antibody labeling. A,B) Low- (A) and high-(B) magnification immunofluorescence images of d18 hiPSC-CMs grown in sparse cultures for 5 days on fibronectin-coated dishes and stained for vimentin (Vim), Nkx2.5, cardiac Troponin T (cTnT), and DAPI. Note that only the nuclei in $cTnT^{+}$ cardiomyocytes, but not in Vim^{+} non-myocytes, are $Nkx2.5^{+}$. Scale bar A) 100µm, B) 25µm.

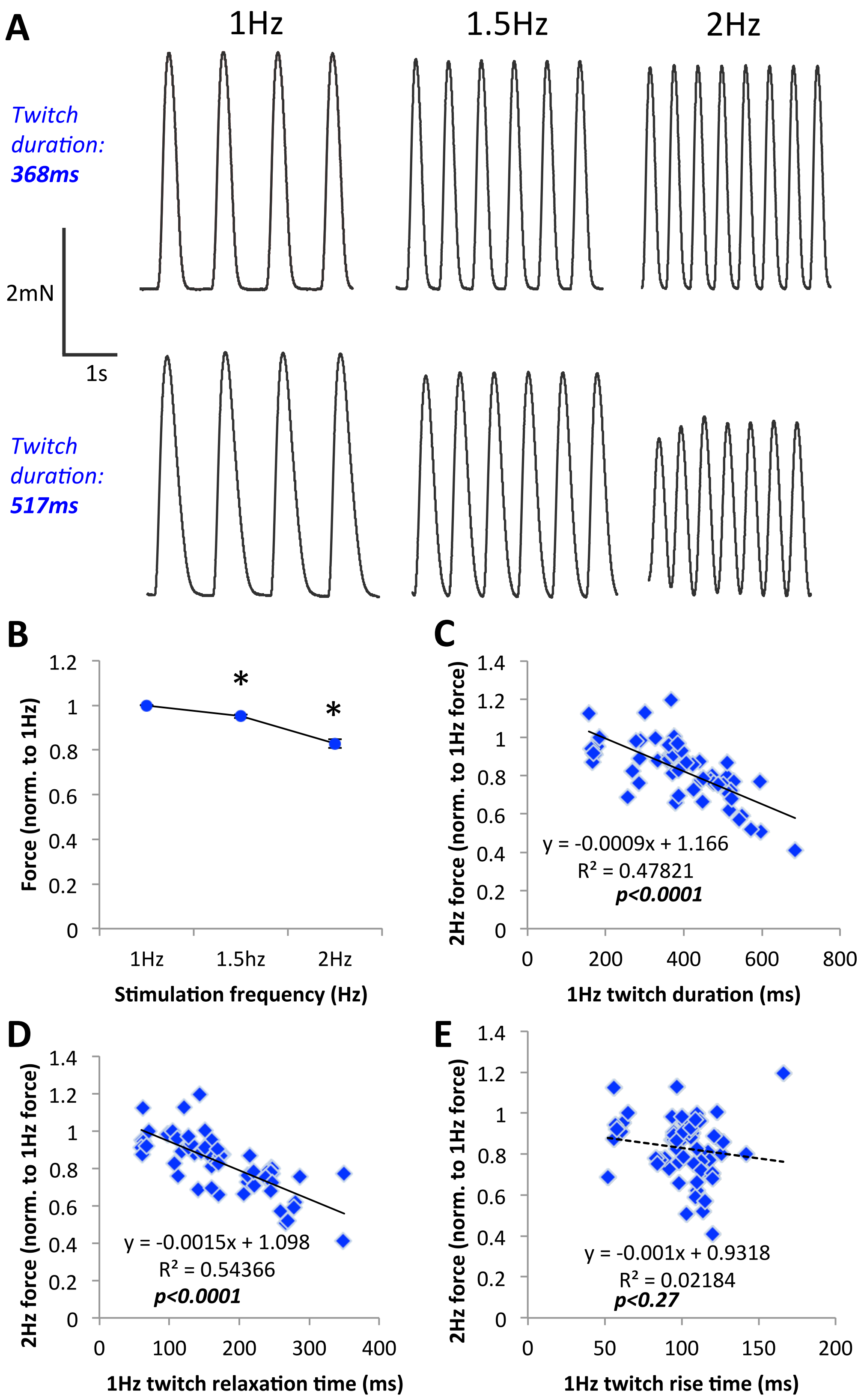

Supplementary Figure 5. Force-frequency relationship in cardiopatches. A) Representative force traces from 3wk old cardiopatches with a shorter (top) and longer (bottom) twitch duration paced at increasing frequencies (1Hz, 1.5Hz, and 2Hz). B) Average force-frequency relationship in N=61 cardiopatches from >20 differentiations performed using multiple hPSC lines (H9, iPSC), differentiation protocols and culture conditions; *p<0.01 vs. 1Hz, paired t-test. C-E) Active force at 2Hz stimulation (normalized to 1Hz force) as a function of 1Hz twitch duration (C), relaxation time (D), and rise time (E); p-values for linear regression testing for slope = 0. Data in B presented as mean ± SEM.

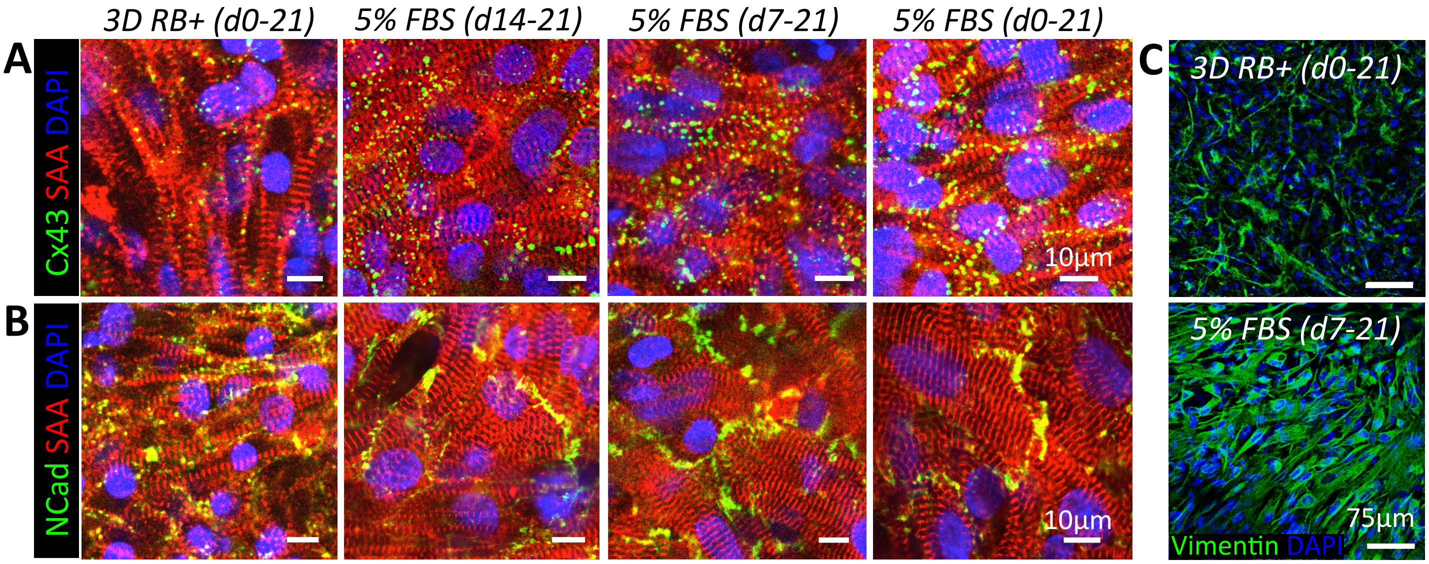

Supplementary Figure 6. Effect of culture media on cardiomyocytes and non-myocytes. A-C) Representative images of cardiomyocytes expressing Connexin-43 (A) and N-cadherin (B) junctions and peripheral layer of non-myocytes expressing vimentin (C) in 3wk old cardiopatches cultured for 3wks in 3D RB+ medium or 1, 2, and 3wks in 5% FBS medium. Scale bar A-B) 10µm, C) 75µm.

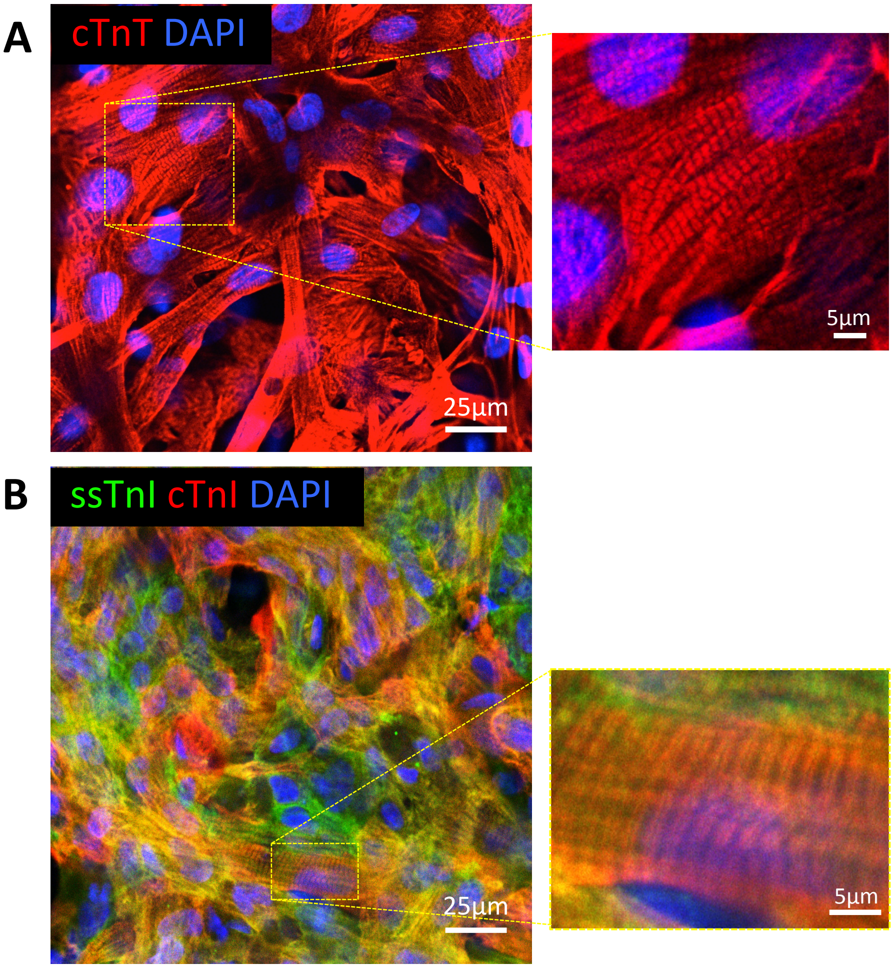

Supplementary Figure 7. Expression of cardiac troponins in cardiopatches. A-B) Representative images of 3wk old cardiopatches stained for cardiac troponin T (A), slow skeletal troponin I (ssTnI, B), and cardiac troponin I (cTnI, B). Scale bar A-B) 25µm, both insets 5µm.

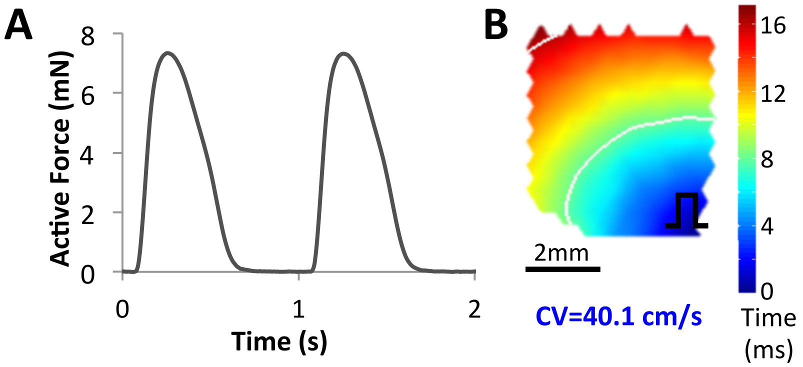

Supplementary Figure 8. **Maximum observed electromechanical function in 0.5MM 7x7 mm patches.** A-B) Examples of highest observed active forces (>7 mN, trace shown in A) and conduction velocities (>40 cm/s, activation isochrone map shown in B) in 3wk old cardiopatches.

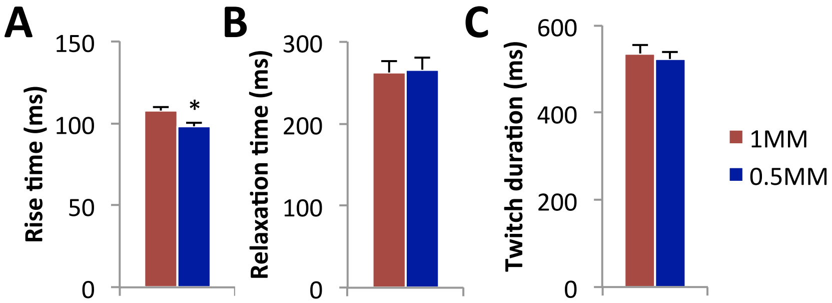

Supplementary Figure 9. **Kinetics of active force generation in cardiopatches.** A-C) Average rise time (A), relaxation time (B), and twitch duration (C) of generated twitch force from 3wk old 1MM and 0.5MM cardiopatches; n=27/30 patches (1/0.5MM) from 9-10 differentiations; *p=0.0055, unpaired t-test. Data presented as mean ± SEM.

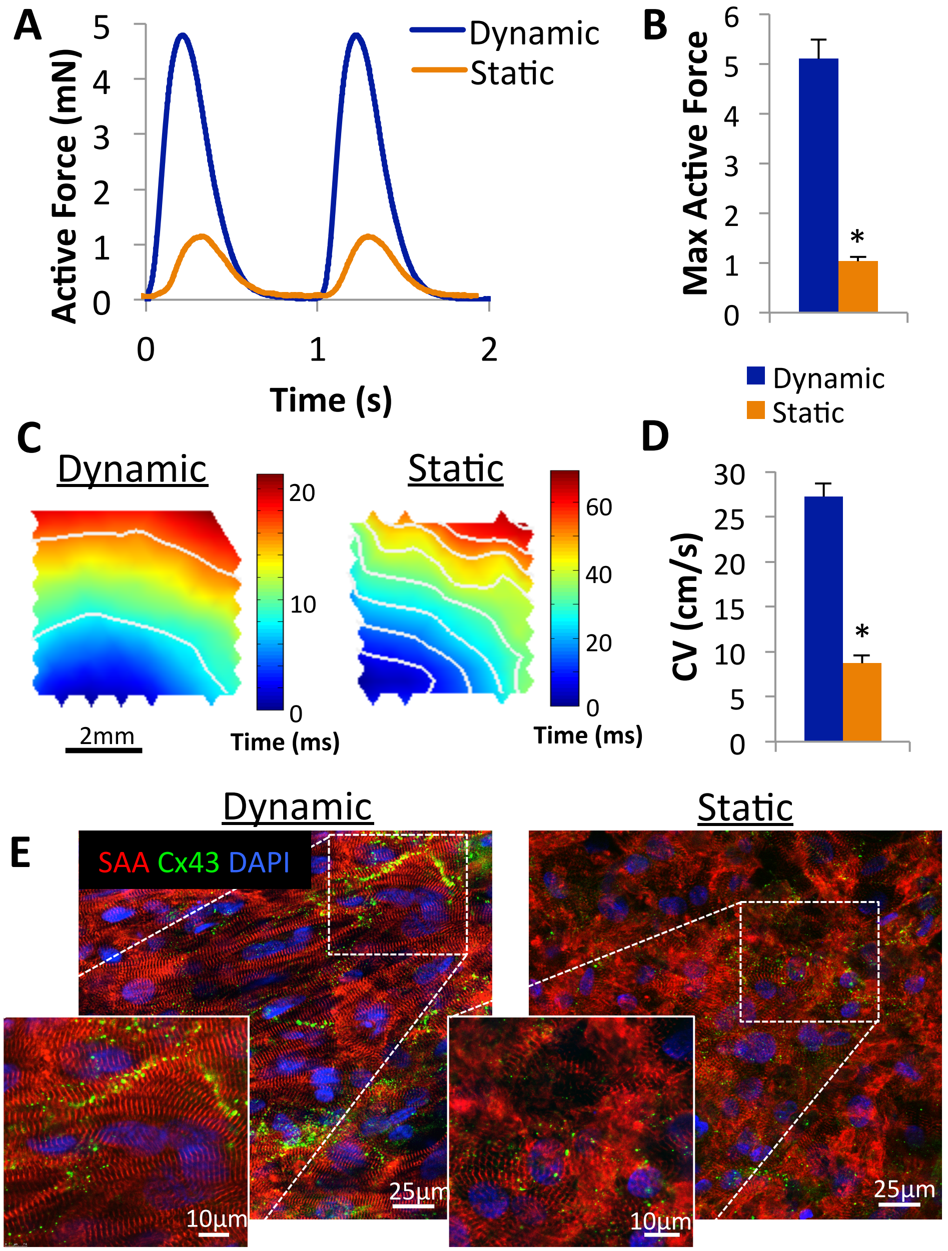

Supplementary Figure 10. Effect of dynamic vs. static culture on structure and functional of 0.5MM cardiopatches. A-B) Representative force traces (A) and average max active force (B) generated by 3wk-old 0.5MM cardiopatches cultured under dynamic (rocker) or static conditions. C-D) Representative isochrone maps (C) and average CV (D) of 3wk-old dynamically or statically cultured 0.5MM cardiopatches. E) Representative confocal images of the two groups immunostained for SAA and Cx43. n=8 patches per group from 2 differentiations; *p<0.0001, unpaired t-test. Data presented as mean ± SEM. Scale bar C) 2mm, E) 25µm (insets 10µm).

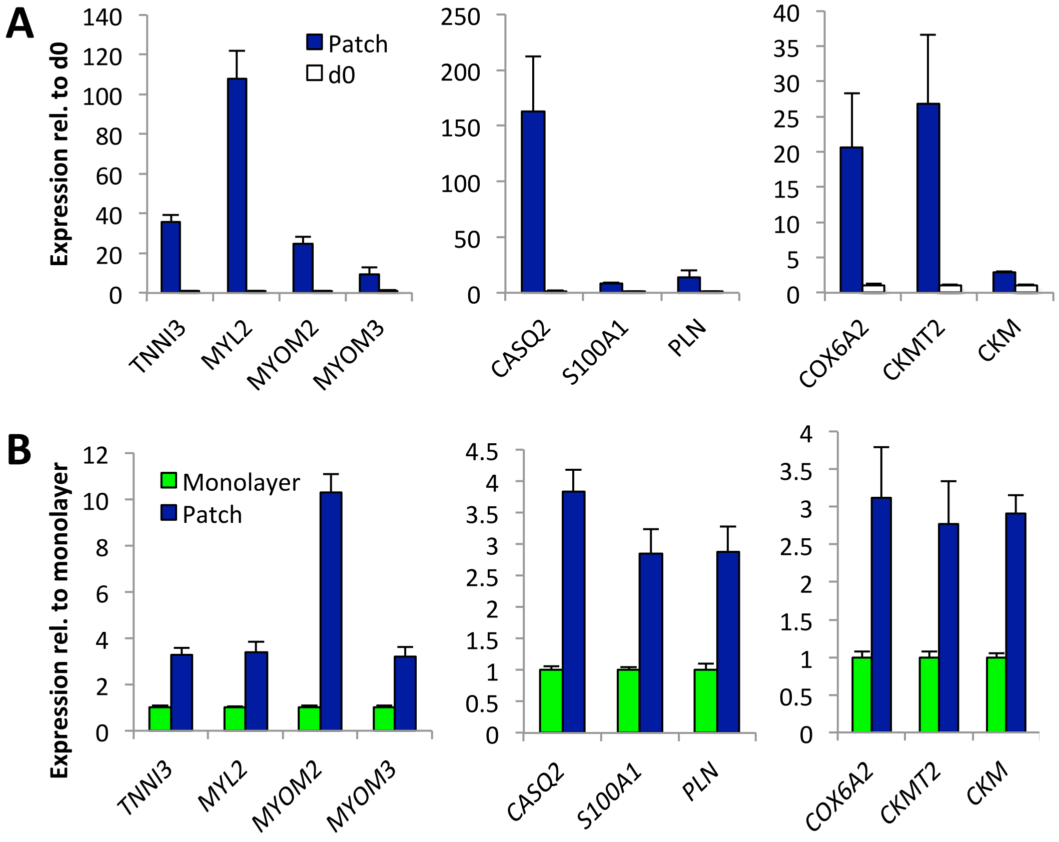

Supplementary Figure 11. Gene expression in cardiopatches. A-B) Gene expression of 3wk old cardiopatches relative to d0 hiPSC-CMs (A) and age-matched 2D monolayers (B); n=6 cardiopatches/monolayers each from 2 independent differentiations. $p<0.0001$ for all groups in patch vs. d0 and patch vs. monolayer, post-hoc Tukey's test following two-way ANOVA. Data presented as mean ± SEM.

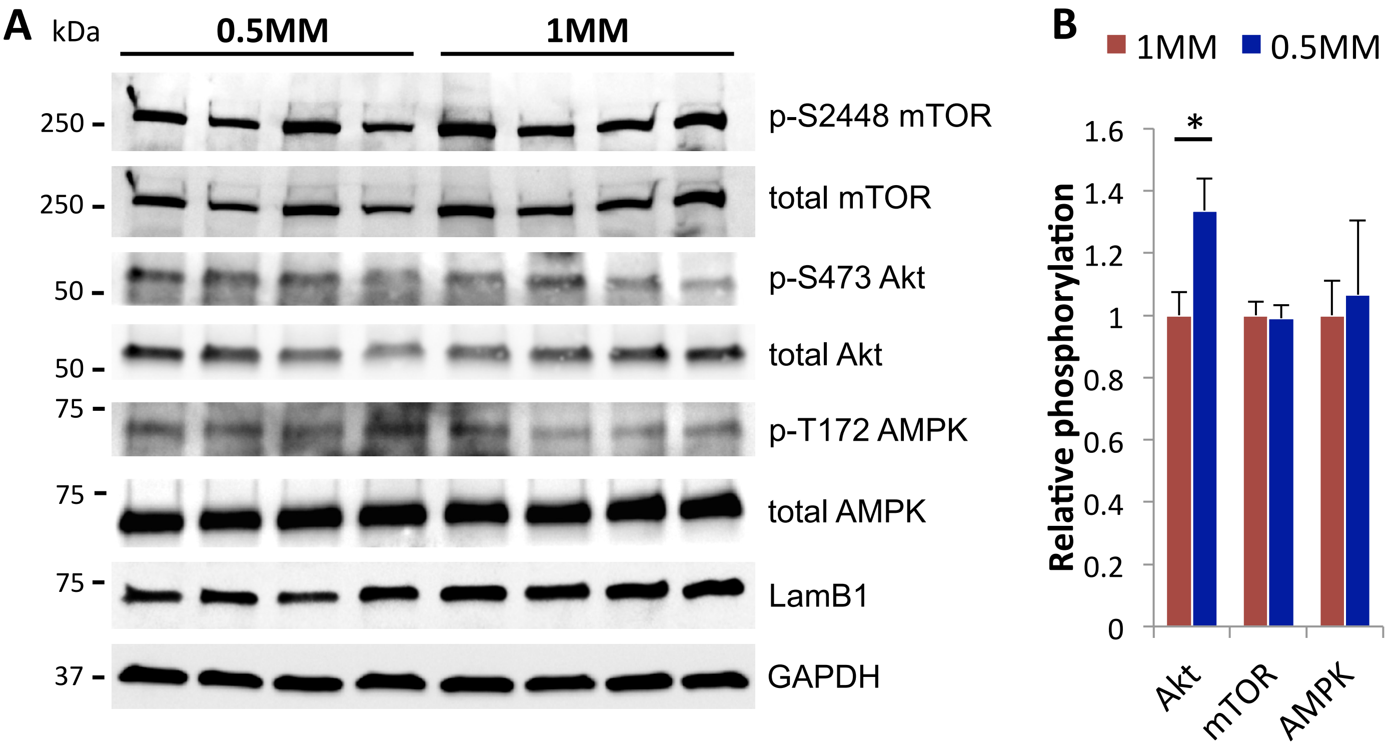

Supplementary Figure 12. **Signaling underlying cell hypertrophy observed in 0.5MM vs. 1MM cardiopatches.** A) Representative Western blots for phosphorylated (Ser2448) and total mTOR, phosphorylated (Ser473) and total Akt, phosphorylated (Thr172) and total AMPK, LamB1 (nuclear envelope) and GAPDH (loading control) in 3wk old cardiopatches generated from 0.5MM or 1MM cells. B) Quantification of relative phosphorylation of Akt, mTOR, and AMPK normalized to 1MM cardiopatches; n=4 cardiopatches per group, *$p<0.05$, unpaired t-test. Data presented as mean ± SEM.

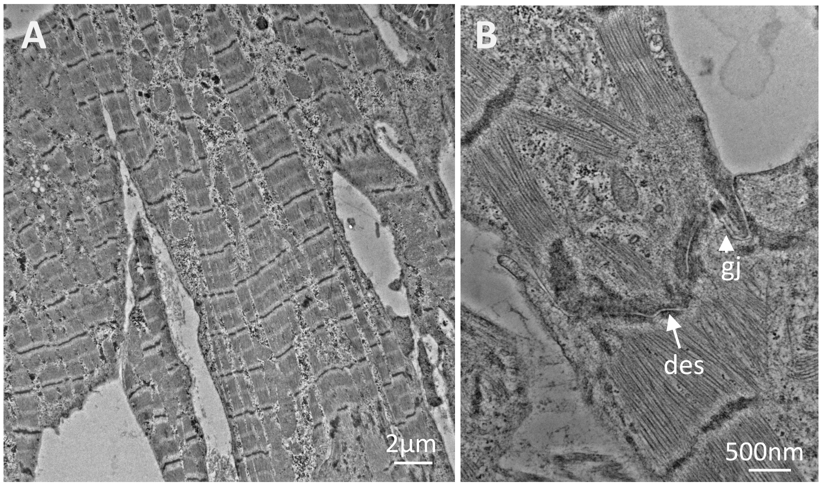

Supplementary Figure 13. hiPSC-CM ultrastructure in cardiopatches. A) Representative low-magnification view of sacromeric structures in 3wk old 0.5MM cardiopatches. B) Localization of gap junctions (gj) and desmosomes (des) within intercalated discs of 3wk old cardiopatches. Scale bar A) 2µm, B) 500nm.

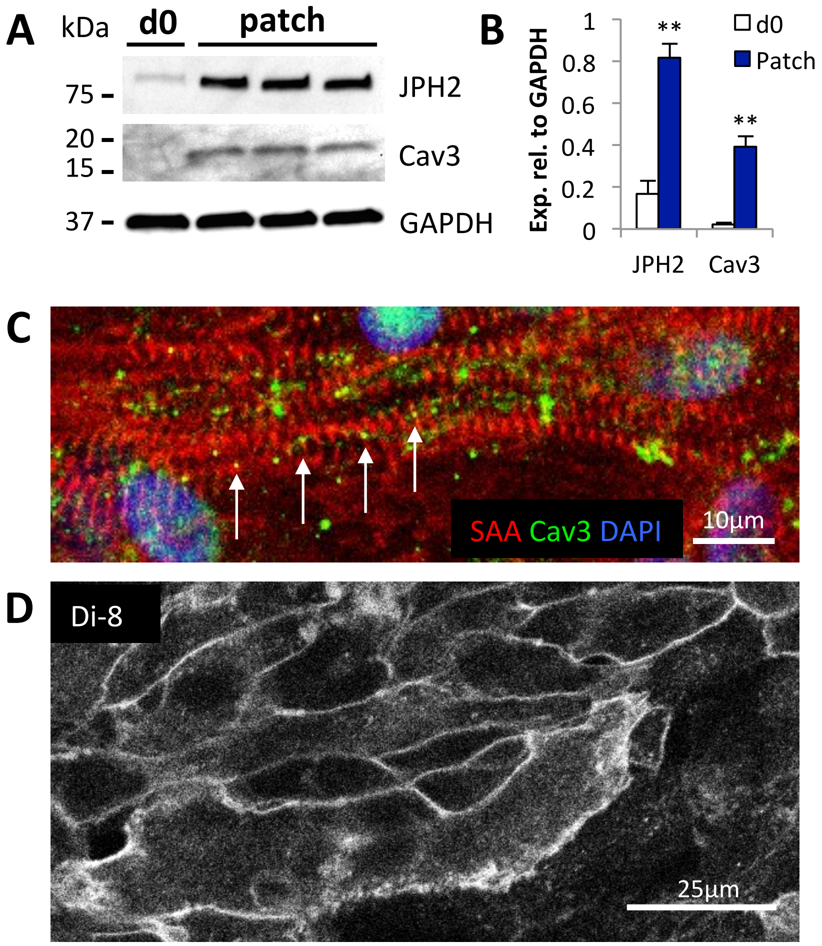

Supplementary Figure 14. T-tubulogenesis in cardiopatches. A) Western blots demonstrating expression of T-tubule-associated proteins Junctophilin-2 (JPH2) and Caveolin-3 (Cav3) in 3wk-old 0.5MM cardiopatches (patch) and input cell populations (d0). B) Quantified JPH2 and Cav3 levels are significantly higher in 3wk-old cardiopatches than input cell population (d0); n=2 input cell populations (d0), n=5 cardiopatches from 2 independent differentiations; **p<0.01, post-hoc Tukey's test. C) Representative confocal image of a 3wk old cardiopatch immunostained for SAA and Cav3; arrows indicate evidence for punctate Cav3 staining co-localizing with SAA striations. D) Live-cell confocal image of a 3wk-old cardiopatch labeled with di-8-ANEPPS does not demonstrate robust, cross-striated T-tubular structures, characteristic of adult myocardium. Data in B presented as mean ± SEM. Scale bar C) 10µm, D) 25µm.

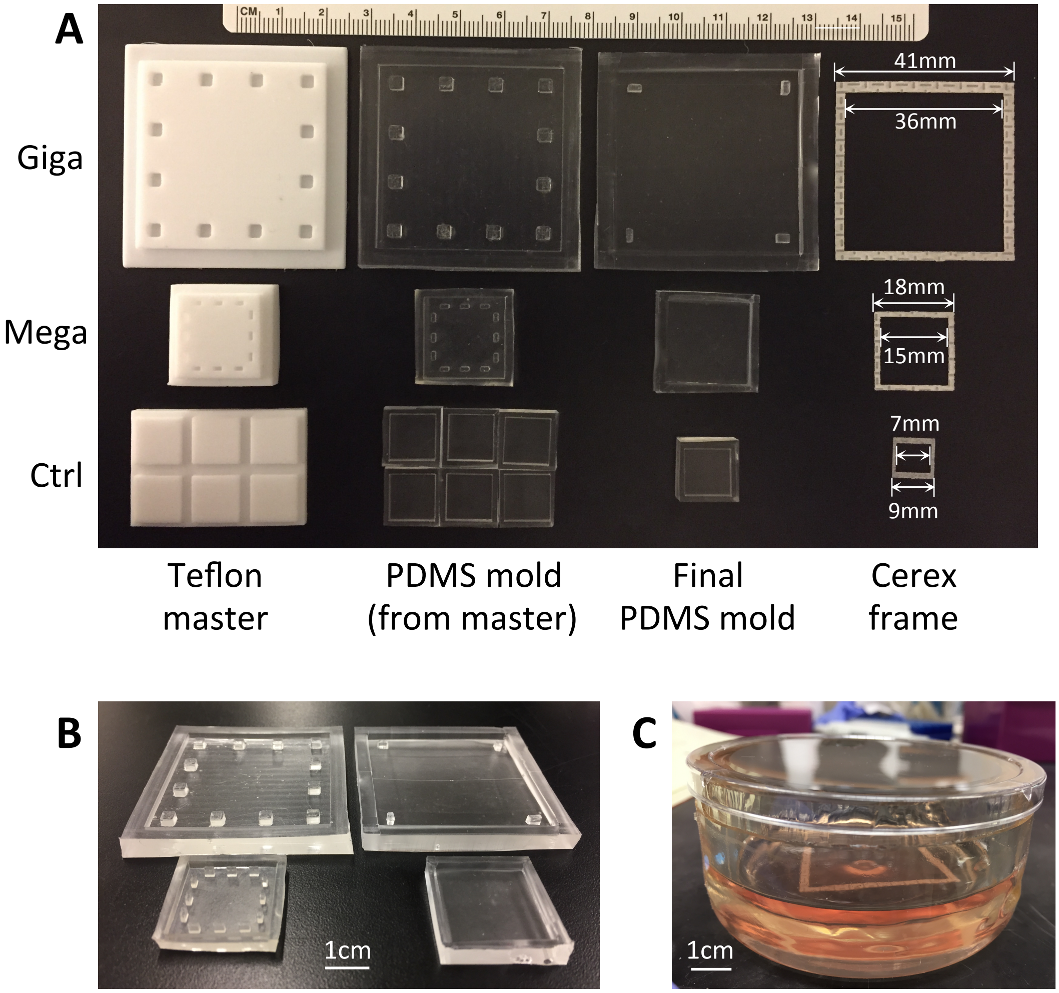

Supplementary Figure 15. Fabrication and culture of scaled-up Mega and Giga cardiopatches. A) Comparison of Teflon masters, PDMS molds and Cerex frames used for generation of control (Ctrl), Mega, and Giga cardiopatches. B) Giga (top) and Mega (bottom) PDMS molds with 12 posts (left) for eventual implantation or minimal posts (right) for maximum tissue coverage while maintaining fixed position of the Cerex frame during hydrogel polymerization. C) Custom-built high-walled PDMS chamber with lid for dynamic culture of Giga patches. Scale bar B-C) 1cm.

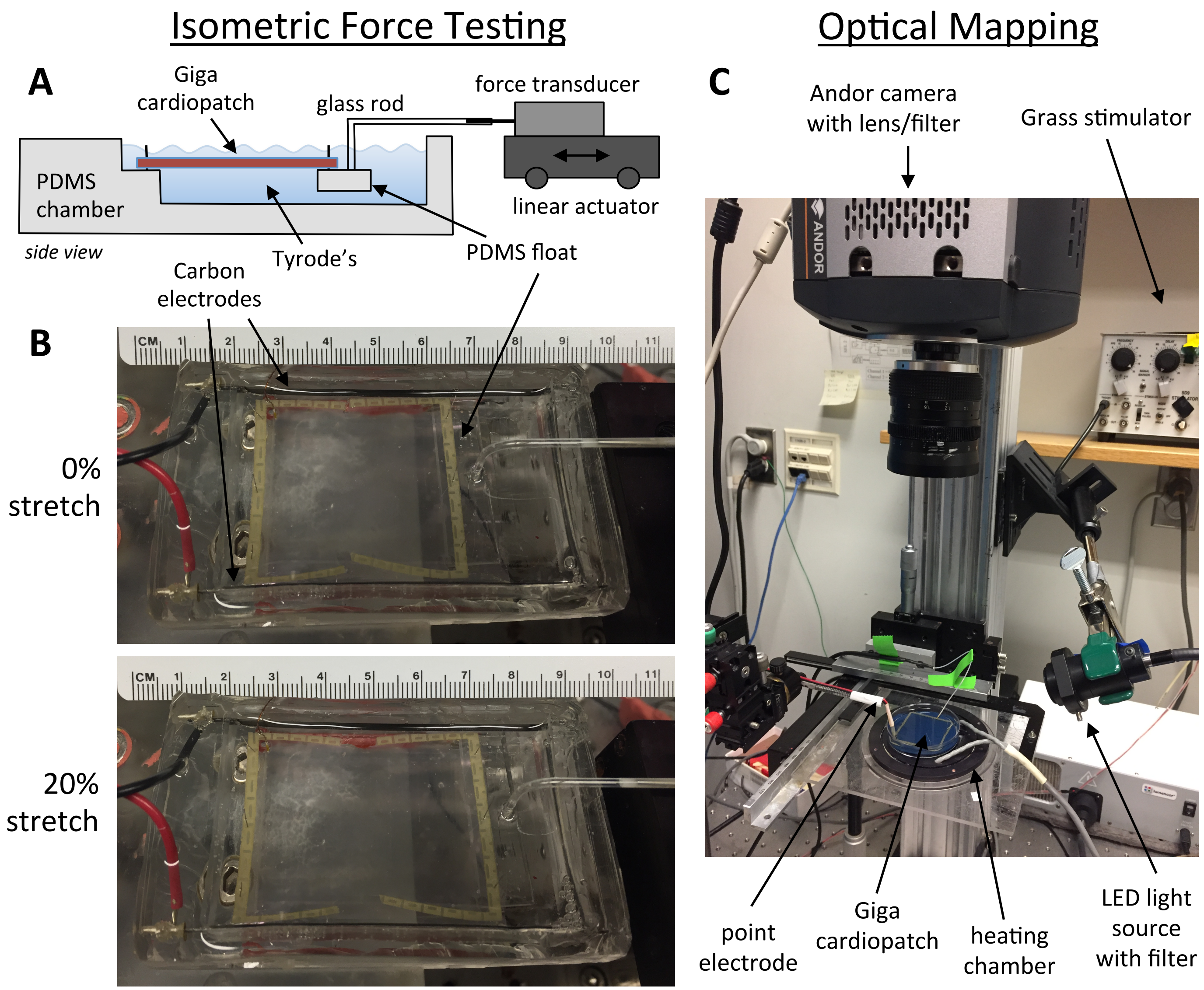

Supplementary Figure 16. Electromechanical testing of Giga cardiopatches. A) Side view schematic of isometric force testing setup. For clarity, field shock electrodes are not depicted. B) Representative views of Giga cardiopatches with two cut sides of the frame at 0% and 20% stretch. C) Setup for optical mapping of Giga cardiopatch using Andor camera with photographic lens and an epi-illumination fluorescence light source.

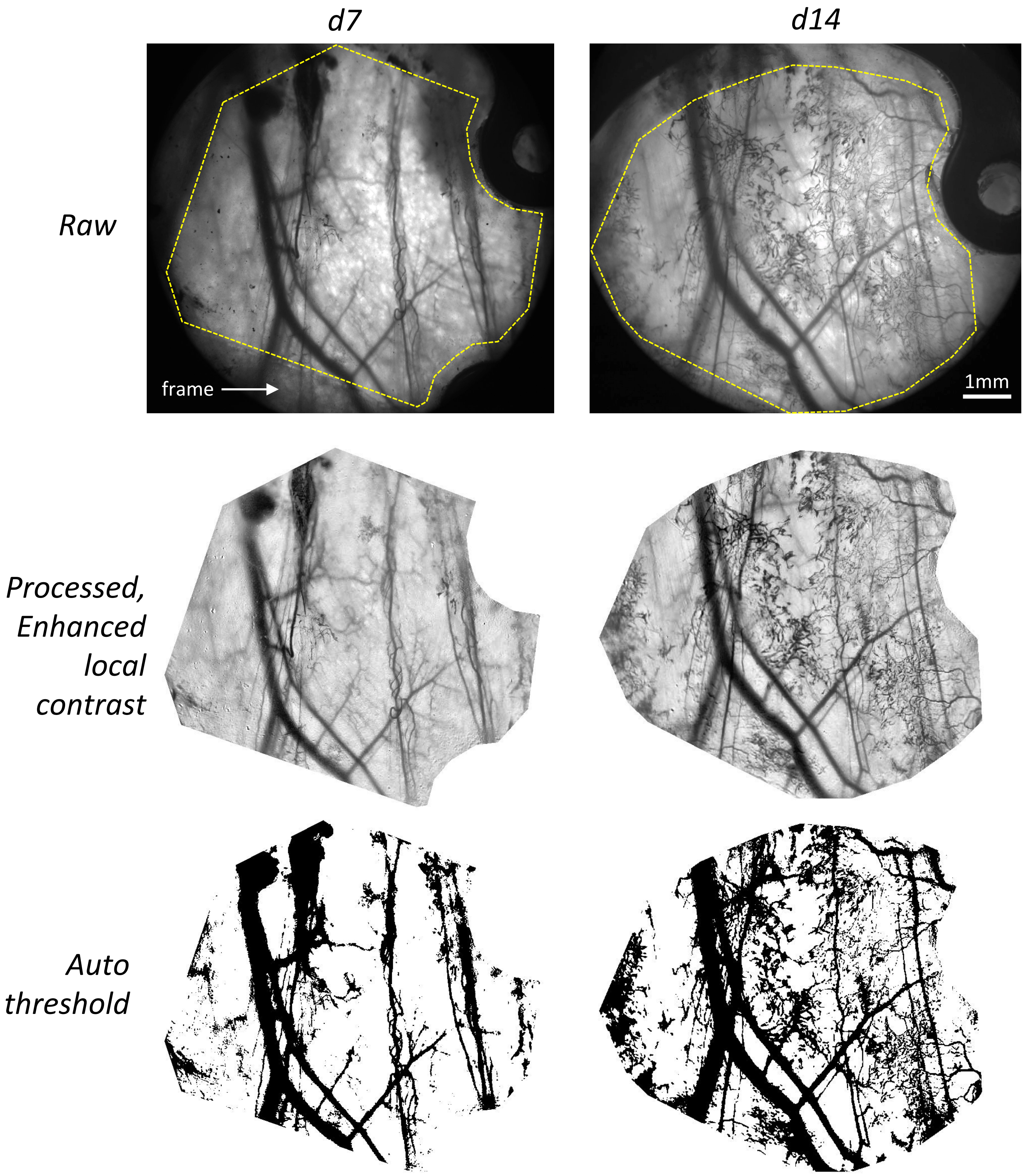

Supplementary Figure 17. **Processing steps for quantification of blood vessel density in window chamber experiments.** Representative raw, processed/contrast enhanced, and thresholded intravitally obtained images used for quantification of blood vessel density in cardiopatches 7d and 14d post-implantation into dorsal window chambers in nude mice. Scale bar 1mm for all panels.

A

Pacing Frequency	Ctrl						Patch					
	1	2	3	4	5	6	1	2	3	4	5	6
2 Hz												
6 Hz								2 sec		3 sec		
8 Hz			sustained									
10 Hz	1 sec							3 sec				
12 Hz				5 sec	1 sec	11 sec		5 sec	17 sec		3 sec	
14 Hz						15 sec		3 sec				
16 Hz						16 sec	5 sec	5 sec				
18 Hz						53 sec			3 sec			
20 Hz	2 sec			29 sec	sustained	4 sec	3 sec	2 sec		4 sec	18 sec	

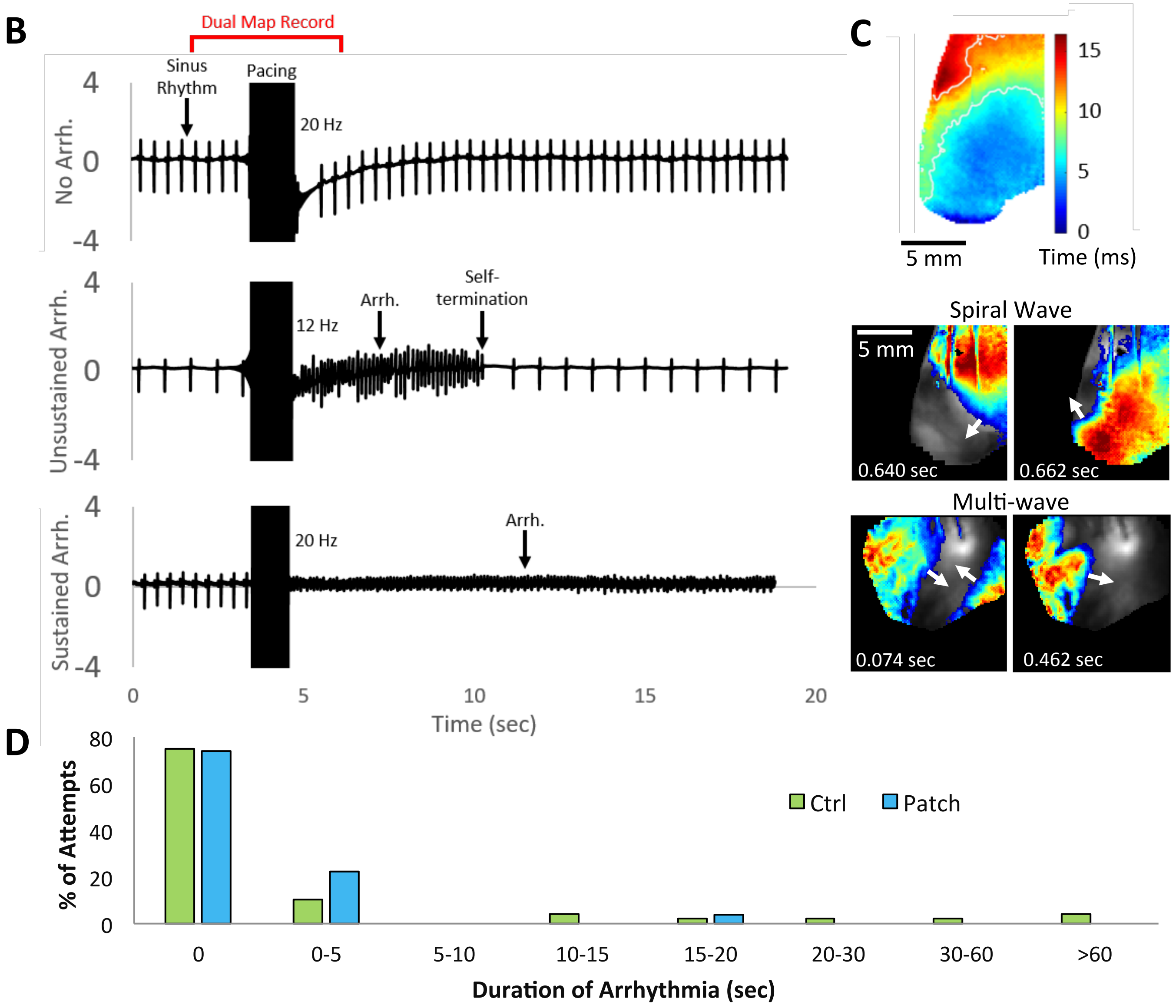

Supplementary Figure 18. Comparison of arrhythmogenicity in cardiopatch-implanted and control rat hearts. A) Summary of all attempts to induce arrhythmias by application of ~1s burst pacing at various frequencies in control (non-implanted) and patch-implanted rat hearts. Attempts shown in white resulted in no induction of arrhythmia and return to sinus rhythm. Attempts resulting in arrhythmias are shown in different levels of red based on the arrhythmia duration. Arrhythmias that did not self-terminate within 1 min were classified as sustained. B) Representative ECG recordings from hearts that after burst pacing returned to sinus rhythm, or underwent short (unsustained) or sustained arrhythmia. C) Representative isochrone map (top) and still-frames from isopotential movies (middle, bottom) from hearts in sinus rhythm (typically displaying epicardial breakthroughs; top) as well as those generating spiral waves (middle) or multi-wave fibrillatory activity (bottom) shortly after pacing; blue to red, rest to peak of propagating action potentials superimposed on grayscale image of the heart. D) Histogram of the percentage of stimulation attempts resulting in arrhythmia of given duration. Scale bar C) 5mm.

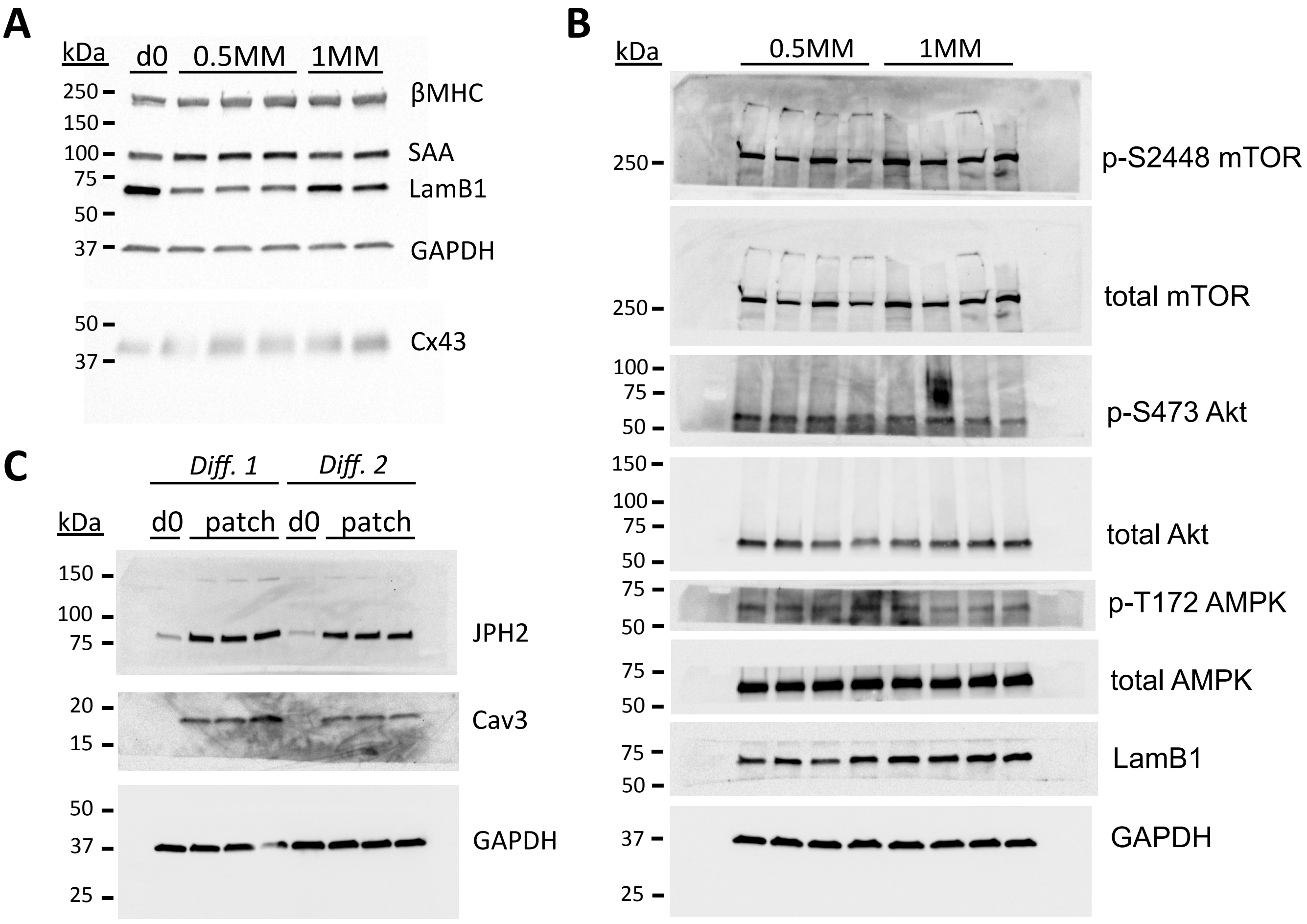

Supplementary Figure 19. **Uncropped Western blot images.** Uncropped Western blot images corresponding to images shown in A) Figure 4D, B) Supplementary Figure 12A, and C) Supplementary Figure 14A.

Supplementary Table 1 - Culture medias used for hPSC-CM cardiopatches

Source	Component	2x cardiac media	3D RB+ media	5% FBS media
(-)	**Basal media**	2x concentrated low glucose DMEM (Thermo 31600-034)*	RPMI-1640 (Sigma R8758 or Thermo 11875)	Low-glucose DMEM (Sigma D6046 or Thermo 11885)
GEHLS SH30071 or SH30396	**Fetal Bovine Serum (FBS)**	(-)	(-)	5%
Thermo 16050	**Horse Serum (HS)**	20%	(-)	(-)
Thermo 17504	**B27 supplement (50x)**	(-)	2%	(-)
Sigma P3032	**Penicillin**	10U/mL	(-)	(-)
Sigma V2876	**Vitamin B12**	4μg/mL	(-)	(-)
Sigma A2504	**Aminocaproic Acid**	2mg/mL	2mg/mL	2mg/mL
Sigma A8960	**Ascorbic Acid 2-Phosphate**	(-)	50μg/mL	50μg/mL
Sigma M6145	**1-thioglycerol**	(-)	0.45μM	0.45μM
Thermo 15140	**Pen/Strep (100x)**	(-)	1%	1%
Thermo 11140	**Non-Essential Amino Acids (100x)**	(-)	1%	1%
Thermo 11360	**Sodium Pyruvate (100x)**	(-)	1%	(-)#

*prepared with half of the recommended volume
#present in basal media
Thermo = ThermoFisher Scientific
GEHLS = GE Healthcare Life Sciences

Supplementary Table 2 – List of primary antibodies

Antibody	Source	Species	Application	Dilution
**Sarcomeric α-actinin (SAA)**	Sigma A7811	Mouse	IF<br>WB	1:200<br>1:1000
**Connexin-43 (Cx43)**	Abcam ab11370	Rabbit	IF<br>WB	1:200<br>1:5000
**N-Cadherin (NCad)**	Abcam ab12221	Rabbit	IF	1:400
**Nkx2.5**	Santa Cruz SC-8697	Goat	IF	1:300
**Ki67**	Abcam ab66155	Rabbit	IF	1:300
**Vimentin (Vim)**	Abcam ab89996	Rabbit	IF	1:500
**Smooth muscle 22α (SM22α)**	Abcam ab10135	Goat	IF	1:200
**β-myosin heavy chain (βMHC)**	Santa Cruz SC-53089	Mouse	WB	1:100
**Cardiac troponin T (cTnT)**	Abcam ab45932	Rabbit	IF<br>FC	1:500<br>1:200
**Cardiac troponin I (cTnI)**	Abcam ab10231	Mouse	IF	1:500
**Slow-skeletal troponin I (ssTnI)**	ProteinTech 22253-1-AP	Rabbit	IF	1:100
**Myosin light chain 2-ventricular (MLC2v)**	ProteinTech 10906	Rabbit	IF	1:200
**Myosin light chain 2-atrial (MLC2a)**	Synaptic Systems 311011	Mouse	IF	1:200
**Lamin B1 (LamB1)**	Abcam ab16048	Rabbit	WB	1:2000
**GAPDH**	Santa Cruz SC-32233	Mouse	WB	1:500
**Phospho-mTOR (Ser2448)**	Cell Signaling Tech. 5536	Rabbit	WB	1:2000
**Total mTOR**	Cell Signaling Tech. 2983	Rabbit	WB	1:2000
**Phospho-Akt (Ser473)**	Cell Signaling Tech. 4060	Rabbit	WB	1:2000
**Total Akt**	Cell Signaling Tech. 4691	Rabbit	WB	1:2000
**Phospho-AMPK (Thr172)**	Cell Signaling Tech. 2535	Rabbit	WB	1:1000
**Total AMPK**	Cell Signaling Tech. 5832	Rabbit	WB	1:1000
**CD31**	Abcam ab28364	Rabbit	IF	1:50
**CD31-APC**	Miltenyi Biotec 130-098-174	Mouse	FC	1:50

**Von Willebrand Factor (vWF)**	Abcam ab6994	Rabbit	IF	1:400
**Junctophilin-2 (JPH2)**	Santa Cruz sc-377086	Mouse	WB	1:300
**Caveolin-3 (Cav-3)**	Abcam ab2912	Rabbit	WB<br>IF	1:200<br>1:20

IF: Immunofluorescence; WB: Western blot; FC: Flow cytometry

Supplementary Table 3 – List of qPCR primers

Gene	Product		Primer
GAPDH	Glyceraldehyde 3-phosphate dehydrogenase	for:	GGAGCGAGATCCCTCCAAAAT
		rev:	GCAAATGAGCCCCAGCCTTC
S100A1	S100 Ca^{2+} binding protein A1	for:	TTCCTGGATGCCCAGAAGGATG
		rev:	CCGTCTCCATTCTCGTCTAGC
MYOM2	Myomesin 2	for:	GCTTTTGCAGAGAAGAATCGTG
		rev:	CATGCTGACGTACTTGGCCT
MYOM3	Myomesin 3	for:	TGAAGCCAGTCTTTGCTCGT
		rev:	ACCTCAGTAGGCTCCCATCT
CKMT2	Creatine kinase, mitochondrial 2	for:	GCCAGGGGAATCTGGCATAA
		rev:	CCCAGCCTCGTTCTTGGATT
CKM	Creatine kinase, muscle	for:	AACCTCAAGGGTGGAGACGA
		rev:	ACTTCCCTTTGAACTCGCCC
COX6A2	Cytochrome c oxidase subunit VIa polypeptide 2	for:	ACTCCTATCTCCACTCGGGC
		rev:	GGTAGGGCTTGGTGCGGAT
CASQ2	Calsequestrin-2	for:	TTGCCATCCCCAACAAACCT
		rev:	AGAGTGGGTCTTTGGTGTTCC
MYL2	Myosin regulatory light chain 2, ventricular isoform (MLC-2v)	for:	TTGGGCGAGTGAACGTGAAAA
		rev:	TCCGCTCCCTTAAGTTTCTCC
TNNI3	Cardiac troponin I (cTnI)	for:	CCTCACTGACCCTCCAAACG
		rev:	GAGGTTCCCTAGCCGCATC
PLN	Phospholamban	for:	GCTGCCAAGGCTACCTAAAAG
		rev:	GACGTGCTTGTTGAGGCATTT

Supplementary Table 4 – Maturation Gene Expression Ratios*

Gene	Adult:fetal	Fetal:hPSC-CM	Adult:hPSC-CM	Ref
**TNNI3**	2	9.3 - 20.8	18.1 - 40.5	1
	3	4.5	13.5	2
	1.4	4(late) – 13.2(early)	5.5(late) – 17.4(early)	3
			22.8(late) – 27.7(early)	4
**MYL2**	1.7	106-122	178-205	1
	1.8	23.4	42.4	2
	1.2	4(late) – 16.3(early)	4.5(late) - 18.6(early)	3
			1.1(late) – 189(early)	4
**MYOM2**	4.1	11.6-19.7	47.6-80.8	1
	4.9	2.7	13.2	2
	1.9	4(late) – 7.2(early)	7.4(late) - 13.4(early)	3
			45(late) – 201(early)	4
**MYOM3**	11.8	3.2-3.7	37.6-43.4	1
	14.1	56.4	797	2
	10.2	2.4(early or late)	24.4(early or late)	3
**CASQ2**	1.2	57.9-105	71.6-130	1
	1.9	32.1	61.6	2
	1.1	7.9(late) – 64.1(early)	8.9(late) - 72.4(early)	3
			291(late) – 670(early)	4
**S100A1**	10.2	1.1-1.2	11.9-12	1
	8.6	1(late) – 1.3(early)	8.7(late) - 11.3(early)	3
			148 (early or late)	4
**PLN**	2	0.6	1.3	2
	1.2	1(late) – 1.3(early)	1.2 - 1.6(early)	3
			1(late) – 3(early)	4
**COX6A2**	2.9	5.6-6.9	16.4-20.1	1
	3	5.8	17.3	2
	1.4	4.1(late) – 5.5(early)	5.8(late) – 7.7(early)	3
**CKMT2**	1.9	16.8-39	32.3-75.1	1
	3	28.7	88.8	2
	1.7	2.3(late) – 7.1(early)	4(late) – 12(early)	3
**CKM**	1.4	7.4-19.2	10.7-27.8	1
	2	4.8	9.4	2
	1.3	2.4(late) - 6(early)	3.2(late) – 7.8(early)	3

Legend:
<2
2-5
5-10
10-20
20-100
100-800

X:Y expression ratios reported as ratio of expression in X relative to that in Y. Fetal and adult refer to human heart samples. Early (<4wks) or late (>4wks) refer to differentiation stage of hPSC-CMs.

References

1 Gupta, M. K. *et al.* Global transcriptional profiles of beating clusters derived from human induced pluripotent stem cells and embryonic stem cells are highly similar. *BMC Dev Biol* **10**, 98, doi:10.1186/1471-213X-10-98 (2010).

2 Xu, X. Q., Soo, S. Y., Sun, W. & Zweigerdt, R. Global expression profile of highly enriched cardiomyocytes derived from human embryonic stem cells. *Stem Cells* **27**, 2163-2174, doi:10.1002/stem.166 (2009).

3 Synnergren, J., Ameen, C., Jansson, A. & Sartipy, P. Global transcriptional profiling reveals similarities and differences between human stem cell-derived cardiomyocyte clusters and heart tissue. *Physiol Genomics* **44**, 245-258, doi:10.1152/physiolgenomics.00118.2011 (2012).

4 Piccini, I., Rao, J., Seebohm, G. & Greber, B. Human pluripotent stem cell-derived cardiomyocytes: Genome-wide expression profiling of long-term in vitro maturation in comparison to human heart tissue. Genom Data 4, 69-72, doi:10.1016/j.gdata.2015.03.008 (2015).
