# Supplementary material for: Cardiopatch platform enables maturation and scale-up of human pluripotent stem cell-derived engineered heart tissues
Source: Nat Commun. 2017 Nov 28;8:1825. doi: 10.1038/s41467-017-01946-x (PMC5705709; doi:10.1038/s41467-017-01946-x)
Supplement: Supplementary file 3 — Description of Additional Supplementary Files [file 41467_2017_1946_MOESM3_ESM.pdf]

Description of Additional Supplementary Files

File Name: Supplementary Movie 1
Description: Representative live views of differentiated hiPSC-CMs following metabolic selection and replating (d15). Scale bar 100µm.

File Name: Supplementary Movie 2
Description: Spontaneous beating of a 7x7mm hiPSC-CM cardiopatch in a 12-well plate after 3wks of 3D culture.

File Name: Supplementary Movie 3
Description: Representative optical mapping recording depicting action potential propagation of 3wk old hiPSC-CM cardiopatch following electrical point-stimulation from the bottom-left corner. CV = 24.8 cm/s. Scale bar 4mm.

File Name: Supplementary Movie 4
Description: 3D confocal reconstruction of a 250µmx250µm region of a 3wk old 0.5MM cardiopatch stained for gap junctional Cx43 (green), sarcomeric α-actinin (red) and DAPI nuclei (blue) across its entire thickness. Scale bar 50µm.

File Name: Supplementary Movie 5
Description: Dynamic culture of a spontaneously contracting 3wk-old Giga cardiopatch within custom PDMS chambers on top of a rocker.

File Name: Supplementary Movie 6
Description: Spontaneous and electrically stimulated (1Hz) contractions of 3wk old hiPSC-CM Giga cardiopatches prior to isometric force measurement.

File Name: Supplementary Movie 7
Description: Comparison of optical mapping recordings from 3wk-old ctrl, Mega and Giga cardiopatches following electrical point stimulation from a corner. CVs = 29.1 cm/s (ctrl), 28.8 cm/s (Mega), and 30.1 cm/s (Giga).

File Name: Supplementary Movie 8
Description: - Intravital imaging of blood flow through newly formed vasculature within implanted hiPSC-CM cardiopatches after 2wks in the dorsal window chamber. Scale bar 200µm.

File Name: Supplementary Movie 9
Description: Intravital gCaMP6 imaging of spontaneous Ca2+ transients of implanted hiPSC-CM cardiopatches after 2wks in the dorsal window chamber. Scale bar 1mm.

File Name: Supplementary Movie 10
Description: Spontaneous and pacing-induced electrical propagation during dual mapping of rat hearts and implanted cardiopatches. Red (voltage, di-4) and green (Ca2+, gCaMP6) wavefronts demonstrate electrical propagation of host heart and implanted cardiopatches, respectively. Traces at the bottom show representative gCaMP6 (green) and di4 (red) signals from a single recording channel with yellow line denoting point in time corresponding to the instant of the movie snapshot. Exogenous pacing from the top was applied for the 1st and 3rd red pulses.

File Name: Supplementary Movie 11
Description: Examples of burst pacing outcomes in patch-implanted and control hearts including return to sinus rhythm, unsustained spiral wave arrhythmia, and sustained multiwave fibrillatory arrhythmia. Blue-to-red represent rest-to-peak of propagating action potentials superimposed on grayscale image of the heart.
